# Large-scale survey reveals pervasiveness and potential function of endogenous geminiviral sequences in plants

**DOI:** 10.1093/ve/veaa071

**Published:** 2020-09-21

**Authors:** Vikas Sharma, Pierre Lefeuvre, Philippe Roumagnac, Denis Filloux, Pierre-Yves Teycheney, Darren P Martin, Florian Maumus

**Affiliations:** URGI, INRAE, Université Paris-Saclay, Plant Breeding Division, 78026, Versailles, France; Forschungszentrum Jülich GmbH, Institute for Bio- and Geosciences 1, IBG1, 52425 Jülich, Germany; CIRAD, UMR PVBMT, Department of Biological Systems, F-97410 St Pierre, La Réunion, France; CIRAD, BGPI, Department of Biological Systems, 34398 Montpellier CEDEX 5, France; BGPI, INRAE, CIRAD, Institut Agro, Univ Montpellier, Department of Biological Systems, 34398 Montpellier CEDEX 5, France; CIRAD, BGPI, Department of Biological Systems, 34398 Montpellier CEDEX 5, France; BGPI, INRAE, CIRAD, Institut Agro, Univ Montpellier, Department of Biological Systems, 34398 Montpellier CEDEX 5, France; CIRAD, UMR AGAP, Department of Biological Systems, F-97130, Capesterre Belle-Eau, Guadeloupe, France; AGAP, Univ Montpellier, CIRAD, INRAE, Institut Agro, Department of Biological Systems, F-97130 Capesterre Belle-Eau, Guadeloupe, France; Division of Computational Biology, Department of Integrative Biomedical Sciences, Institute of infectious Diseases and molecular Medicine, University of Cape Town, OBSERVATORY 7925 Cape Town, South Africa; URGI, INRAE, Université Paris-Saclay, Plant Breeding Division, 78026, Versailles, France

**Keywords:** *Geminiviridae*, endogenous virus, *Viridiplantae*, genomes, transcriptomes, phylogeny, paleovirology

## Abstract

The family *Geminiviridae* contains viruses with single-stranded DNA genomes that have been found infecting a wide variety of angiosperm species. The discovery within the last 25 years of endogenous geminivirus-like (EGV) elements within the nuclear genomes of several angiosperms has raised questions relating to the pervasiveness of EGVs and their impacts on host biology. Only a few EGVs have currently been characterized and it remains unclear whether any of these have influenced, or are currently influencing, the evolutionary fitness of their hosts. We therefore undertook a large-scale search for evidence of EGVs within 134 genome and 797 transcriptome sequences of green plant species. We detected homologues of geminivirus replication-associated protein (Rep) genes in forty-two angiosperm species, including two monocots, thirty-nine dicots, and one ANITA-grade basal angiosperm species (*Amborella trichopoda*). While EGVs were present in the members of many different plant orders, they were particularly common within the large and diverse order, *Ericales*, with the highest copy numbers of EGVs being found in two varieties of tea plant (*Camellia sinensis*). Phylogenetic and clustering analyses revealed multiple highly divergent previously unknown geminivirus Rep lineages, two of which occur in *C.sinensis* alone. We find that some of the *Camellia* EGVs are likely transcriptionally active, sometimes co-transcribed with the same host genes across several *Camellia* species. Overall, our analyses expand the known breadths of both geminivirus diversity and geminivirus host ranges, and strengthens support for the hypothesis that EGVs impact the biology of their hosts.

## 1. Introduction

Geminiviruses infect a wide array of important crop species and are considered the most economically important group of plant viruses in the tropical and subtropical regions of the world (Rojas et al. 2018). For instance, geminiviral diseases are currently considered the most important biotic constraint on cassava production in Africa, causing estimated annual economic losses of $1.9–2.7 billion and threatening the food security of hundreds of millions of sub-Saharan Africans (Rey and Vanderschuren 2017; Jacobson, Duffy, and Sseruwagi 2018).

Geminiviruses have non-enveloped twinned icosaedral particles that each contains one single-stranded circular genomic DNA molecule of between 2.5 and 3.7 kb. Geminivirus genomes are composed of one or two components that encode four to eight proteins, the most conserved of which is a replication-associated protein (Rep) that is required for the initiation of rolling circle replication (Jeske 2009; Filloux et al. 2015; Varsani et al. 2017). Based on their genome structure, pairwise nucleotide sequence identities, insect vectors, and host plant species, nine genera are currently recognized within the family *Geminiviridae* (*Becurtovirus*, *Begomovirus*, *Capulavirus*, *Curtovirus*, *Eragrovirus*, *Grablovirus*, *Mastrevirus*, *Topocuvirus*, and *Turncurtovirus*). In addition, some apparent, but highly divergent, geminivirus species are currently still unassigned due to missing information on their insect vectors and particle morphologies (Varsani et al. 2017).

Endogenous viral elements (EVEs) are viral sequences that have been integrated into the nuclear genomes of host germline cells, enabling their vertical transmission and potential fixation in host populations (Feschotte and Gilbert 2012; Frank and Feschotte 2017). For viruses such as retroviruses which encode an integrase, endogenization is an active process that is an essential part of the virus life cycle (Silva et al. 2017; Santini et al. 2018). For most other viruses, however, endogenization is a passive process resulting from non-homologous recombination between virus and host genomes, usually in the context of either double-stranded DNA break repair or transposon-mediated processes (Geering et al. 2014). Just like with fossil records, the study of EVEs can illuminate the evolutionary history of virus families. Such studies could also reveal whether EVEs have impacted the evolution of their host species (Carrasco, Sánchez-Navarro, and Elena 2019).

Although most of the plant EVEs that have so far been characterized belong to the family *Caulimoviridae* (Geering et al. 2014; Diop et al. 2018), the first plant EVEs to be discovered were geminivirus-derived sequences in the nuclear genomes of various *Nicotiana* species (Kenton et al. 1995; Bejarano et al. 1996; Ashby et al. 1997; Murad et al. 2004). Since then endogenous geminivirus (EGV) elements have also been identified in the genomes of yam (*Dioscorea* spp.), apple (*Malus domestica*), lettuce (*Lactuca sativa*), cottonwood (*Populus trichocarpa*), and coffee (*Coffea canephora*) (Liu et al. 2011; Martin et al. 2011; Filloux et al. 2015). A feature in common amongst all these EGVs is that they consist of only partial geminivirus genomes and are therefore unlikely to be infectious (Murad et al. 2004; Filloux et al. 2015). However, more recently, EGVs that are transcriptionally active and potentially express functional proteins have been discovered in the genomes of several yam species (*Dioscorea* spp.) (Filloux et al. 2015). At least two genetically distinct geminiviruses likely became integrated into the genome of an ancestral yam species more than 1.4 million years ago (mya). Further, there is evidence that the *rep* genes of at least one of these integrated viruses evolved for a prolonged period post-integration to maintain the amino acid encoding potential of the gene (Filloux et al. 2015).

Here, we have computationally explored publicly available plant genome sequences and plant transcriptomes for the presence of homologues of geminiviral *rep* genes to both determine the pervasiveness and diversity of EGVs, and explore the potential impacts of newly discovered EGVs on the biology and evolution of the plants species in which they occur.

## 2. Materials and Methods

### 2.1 Query Rep sequence library

A Rep protein query library was built using a set of thirty-five sequences representative of the entire breadth of known geminivirus diversity (Varsani et al. 2017) and four representative sequences from genomoviruses (YP_009115508, ASH99142) and phytoplasma plasmids (WP_015083745, P60470).

### 2.2 Subject plant genome and transcriptome database

A total of 134 plant genome sequences including algae, moss, angiosperms, and gymnosperms were downloaded from multiple sources (Supplementary Table S1). Six *Ericales* plant genomes were additionally downloaded for a more thorough search of EGVs within this plant order (Supplementary Table S9). Similarly, a set of 797 plant transcriptome datasets from *Viridiplantae* (green algae to angiosperms) were downloaded from the NCBI FTP site (https://www.ncbi.nlm.nih.gov/Traces/wgs/? page=1&view=TSA) (Supplementary Table S2). We have also used Illumina paired sequencing reads to search for Rep homologues in *Primula veris* genomic data (run SRR1658103).

### 2.3 EGV detection pipeline

tBLASTn searches (Altschul et al. 1990) were performed with a minimum *E*-value threshold of 10^−5^ using a Rep protein query library against the targeted plant genomes and transcriptomes following an approach similar to that defined in a previous study (Diop et al. 2018). Overlapping hits were merged using S-MART (Zytnicki and Quesneville 2011). To recover complete gene sequences, hits to physically clustered genomic loci were extended up-to 1 kb both up- and downstream of the hit sites. Various geminivirus species encode *rep* genes with introns and identification of these genes is challenging using traditional gene prediction programs (Varsani et al. 2017). Thus, all extended genomic loci both with and without introns were translated into amino acid sequences using the Exonerate program (Slater and Birney 2005). To reduce false positives, all predicted amino acid sequences were compared against the viral Rep protein library using BLASTp with default parameters and an *E*-value cut-off of 10^−5^. Query amino acid sequences with the best blast hits against the viral Rep amino acid sequences that were >89 residues in length were included in further analysis. Lastly, the Rep-like amino acid sequences retrieved from each plant genome or transcriptome were clustered at an 80 per cent identity threshold using UCLUST (Edgar 2010). For each of the individual clusters thus obtained the longest sequence was selected as the representative sequence for that cluster and was used for all further analyses.

### 2.4 Sequence alignment and phylogenetic tree construction

To further reduce the number of potential EGV sequences, the identified representative geminivirus-like Rep sequences from each cluster were re-clustered at 70 per cent identity using UCLUST (Edgar 2010). To maximize the quality of multiple sequence alignments and ensure the robustness of subsequent phylogenetic analysis, we selected only those amino acid sequences that were longer than 180 residues in length. In addition to this, Rep amino acid sequences representative of the main known geminivirus groups as well as from the recently sequenced *Camellia chlorotic dwarf-associated virus* genome (Zhang et al. 2018) were included in the dataset.

A multiple amino acid sequence alignment was constructed using MAFFT v7.271 (Katoh and Standley 2014). Poorly aligned sequences were then removed based on gapped columns using the maxalign program (Gouveia-Oliveira, Sackett, and Pedersen 2007). Manual curation was then carried out to improve the quality of the alignment. Regions of the alignment with large numbers of gaps were then trimmed with trimAL 1.2rev59 (Capella-Gutiérrez, Silla-Martínez, and Gabaldón 2009) using the ‘-automated1’ command. A maximum-likelihood (ML) phylogenetic tree was constructed with phyml 3.1 as implemented in Seaview (Gouy, Guindon, and Gascuel 2010) using the VT amino acid substitution model, as selected using Prottest3 3.4.2 (Darriba et al. 2011). The phylogenetic tree was visualized with the R package, ggtree (Yu et al. 2017).

**Similarity-based classification:** Due to the difficulty of combining all the Rep sequences together within a single multiple alignment, we used a BLAST-based similarity approach implemented in the CLANS (CLuster ANalysis of Sequences) program to cluster sequences based on their relationships (Frickey and Lupas 2004). Briefly, the CLANS program measures all-against-all BLASTp pairwise similarities between matching oligopeptide sequences to cluster the queried amino acid sequences. All known and newly identified viral-like Rep amino acid sequences were clustered in CLANS using a Jackknife clustering method with an *E*-value threshold of 10^−20^ and the BLOSUM 62 substitution matrix (Frickey and Lupas 2004). The pairwise similarity groups thus produced were visualized using ggplot2 (Ginestet 2011).

### 2.5 Phylogenetic placement of EGVs in the *Ericales* order

To estimate the number of distinct geminivirus-like sequences that were independently integrated within the genomes of plants within the *Ericales* order, a phylogeny of all EGVs discovered within the genomes of plants within this order, along with a representative set of exogenous geminiviral reference sequences, was constructed. Due to the inherent difficulty of aligning sequences that have potentially undergone duplications, insertions or deletions during their post-integration histories, the analysis was restricted to the catalytic domain of the *rep* gene (AL1 domain, PFAM ID: PF00799). Only sequences containing at least 150 nts of the AL1 domain were retained for further analysis. This requirement excluded the five EGV sequences obtained from the *Phlox* genus from further analysis. The sequence set (1,437 EGV sequences that encode the AL1 domain along with seventy-four exogenous geminivirus reference sequences) was aligned using MAFFT v7.310 (LINSI parameter set) (Katoh and Standley 2014) and the phylogeny was produced using Fasttree 2.1.8 (Price, Dehal, and Arkin 2010) with the GTR + gamma substitution model. The phylogeny was visualized and edited using the R package, ape (Paradis and Schliep 2019). The number of integration events was deduced from this tree by visual inspection, and the integration event number thus inferred should be taken as a lower bound on the number of actual integration events. Specifically, with additional sampling of exogenous geminiviruses and EGVs it will undoubtedly transpire that many of the individual monophyletic sequence clusters that appear to have a plausible common integration origin will be discovered to be non-monophyletic. Put another way, if these clusters are found in the future to contain either exogenous virus sequences or sequences of an endogenous origin from a plant species that is distantly related to the plant species from which the other EGV sequences in the cluster were obtained, this would invalidate a single integration origin hypothesis for all the sequences in those clusters.

### 2.6 Inferring the genome sequences of the viruses that gave rise to EGVs

After cutting the regions of plant genomes surrounding EGV sequences (including 5 kb up- and downstream flanking regions) into a series of k-mers (K21, K33, K55, K77, K99, K127) genome assemblies were attempted with Spades version 3.13.0 (Bankevich et al. 2012) using the command, –only-assembler. Assembled contigs with high coverage that were within the range expected for geminivirus genome size (2.5–5 kb) were considered for further analyses. Putative geminiviral contigs were cut at the conserved nonanucleotide sequence ‘TAATATTAC’ that forms the origin of virion strand replication in the geminiviruses. Open reading frames (ORFs) were identified using prodigal (Hyatt et al. 2010) and NCBI ORF finder (https://www.ncbi.nlm.nih.gov/orffinder/). All identified ORFs larger than seventy-five amino acids in length were functionally annotated using the NCBI non-redundant protein database. Full geminivirus-like genomes were visualized using Unipro UGENE program (Okonechnikov et al. 2012).

### 2.7 Host comparison

In order to identify the known and unknown status of viral host plant species, each identified plant species with geminivirus-like homologues was searched against the Virus-Host Database (http://ftp://ftp.genome.jp/pub/db/virushostdb/old/release94/) (Mihara et al. 2016) and the published literature.

### 2.8 Distribution of high copy-number EGV sequences across plant genomes

Plant genomes presenting high EGV copy numbers (>100 copy, listed in Supplementary Table S1) were used for EGV density distribution analyses. The number of EGVs within 100 kb bins of each plant genome (assuming a single artificial scaffold) was obtained using the ‘make-windows’ and ‘intersect’ commands of the bedtools program (Quinlan and Hall 2010) before visualization in R using the ggplot package (Ginestet 2011). The *P*-value of the genomic clustering of the EGV was estimated using the binoial distribution as implemented in R.

### 2.9 Neighboring genomic content analysis

The flanking region, 5 kb up and downstream of EGVs from genomes, and complete EGV harbouring transcripts were annotated based on the Conserved Domain Database (CDD) against Pfam domains using RPS-BLAST with the parameters -m 8 -e 0.001 -F T (Marchler-Bauer et al. 2015). Comparative domain plots were visualized using the R package ggplot2 (Ginestet 2011).

### 2.10 Co-transcript analyses

EGV/HSP70 co-transcripts from six *Camellia* species were searched for within the NCBI Transcriptome Shotgun Assembly (TSA) database (https://www.ncbi.nlm.nih.gov/genbank/tsa/) using BLASTn. The ten homologous nucleotide transcript sequences with the lowest *E*-scores that were identified were extracted and aligned together using MAFFT v7.271 (Katoh and Standley 2014). ORFs were then predicted for all the transcripts using the online RAST server (Meyer et al. 2008) and annotated using NCBI BLAST. Complete annotated transcripts were aligned and visualized using the program, Easyfig (Sullivan, Petty, and Beatson 2011). HSP70 and Rep sequences were extracted and aligned using MAFFT v7.271 (Katoh and Standley 2014). The aligned protein sequences for each gene with corresponding nucleotide sequences were converted into a codon alignment using the PAL2NAL program (Suyama, Torrents, and Bork 2006). The dN/dS ratios of protein-coding genes were computed from codon alignments using the datamonkey webserver (Weaver et al. 2018) (http://www.datamonkey.org/) implementation of the fast unbiased Bayesian approximation (FUBAR) method (Murrell, 2013)^.^ Sites inferred by FUBAR to be evolving under positive/negative selection with associated posterior probabilities ≧0.9 were reported. 

### 2.11 Methylation analysis

DNA methylation data for *C.sinensis var. assamica* (accession number: GSE119992) was downloaded directly from the Gene Expression Omnibus (GEO) database in WIG format. The WIG file for each methylation context (CG, CHG, and CHH) was converted to BED file format with the command, wig2bed, using the BEDOPS tool (Neph et al. 2012). Protein coding genes for the *C.sinensis var. assamica* were downloaded from the website, www.plantkingdomgdb.com/tea_tree/. Repeated elements or transposable elements (TE) in this genome were predicted using RepeatScout v1.0.5 (with the option stopafter = 500) (Price, Jones, and Pevzner 2005) on a genomic subset comprising the longest contigs from the assembly to a cumulative length of 412 mb. The resulting library was used to annotate the whole assembly using the TEannot pipeline v2.4 with default parameters (Quesneville et al. 2005). The positions of EGVs, protein-coding genes and repeats within the *C.sinensis var. assamica* genome were intersected with those of methylated sites in each sequence context (CG, CHG, and CHH) using bedtools (Quinlan and Hall 2010). All datasets were normalized by the length of the targeted EGVs, coding genes and repeats and used for the comparison of methylation levels. Comparative methylation plots were visualized using the R package, ggplot2 (Ginestet 2011). Significant differences between EGVs, genes, and repeats for each methylation context (CG, CHG, and CHH) were tested for using pairwise Wilcoxon tests as implemented in R.

## 3. Results and discussion

### 3.1 EGV sequences likely occur within a significant proportion of plant genomes

To determine the pervasiveness of EGV sequences within plant genome sequences, we searched within 134 publicly available plant genomes for geminivirus-like encoded Rep protein sequences using a custom designed, tBLASTn-based, EGV discovery pipeline. Putative EGV encoding Rep protein homologues were detected in, and retrieved from, seventeen (12.7%) of the analyzed plant genomes (Supplementary Table S1). The seventeen potentially EGV-containing genomes were all from angiosperms, and included fourteen of the seventy-seven analyzed eudicots, two of the thirty-one analyzed monocots, and the single ANITA-grade basal angiosperm that was analyzed. No EGV was detected in the genomes of any of the five gymnosperm or eight fern species that were analyzed (Supplementary Fig. S1). Incidentally, the reciprocal BLAST step of the discovery pipeline indicated closer relatedness to geminiviruses than to either genomoviruses or phytoplasmal plasmids for all prospective EGVs other than the one identified in *Oryza longistaminata.* This exceptional sequence was found on a 2-kb contig that shared 89.71 per cent identity with Giant panda-associated gemycircularvirus (Zhang et al. 2017); possibly suggesting that it originated from an episomal genomovirus sequence within the plant from which it was obtained. This sequence was disregarded in further analyses.

Besides geminivirus Rep homologues previously reported in the genomes of *Dioscorea alata* (water yam), *Malus domestica* (apple tree), *Lactuca sativa* (lettuce), *Coffea canephora* (coffee), *Populus trichocarpa* (black cottonwood), and *Nicotiana* sp. (*Nicotiana tomentosiformis* and *Nicotiana tabacum*) (Filloux et al. 2015; Murad et al. 2004), we further identified putative geminivirus Rep homologues within the published genomes of *Amborella trichopoda* (Amborella), *Macadamia integrifolia* (macadamia), *Helianthus annuus* (sunflower), *Vaccinium corymbosum* (blueberry), *Vaccinium macrocarpon* (cranberry), *Oryza longistaminata* (longstamen rice), *Olea europaea* (olive), *Solanum melongena* (eggplant), and *Camellia sinensis* (tea tree; in both var. *sinensis* and *assamica*). Overall, out of sixteen plant species with genomes containing EGVs only seven are known hosts of geminiviruses.

In total, we identified geminivirus Rep homologues at 2,359 genomic loci within the different plant genomes with copy numbers per genome ranging from 1 to 967 (Supplementary Table S1). We found that more than 100 copies of the *rep* gene are integrated in the *N.tabacum* (116 copies) and *D.alata* (148 copies) genomes, confirming previously published results (Kenton et al. 1995; Murad et al. 2004; Filloux et al. 2015). Strikingly, the highest copy numbers were observed in the two analyzed *Camellia sinensis* varieties: 967 in var. *sinensis* and 918 in var. *assamica* (Supplementary Table S1).

### 3.2 Some EGV-encoded *rep* genes are likely transcriptionally active

As EGV sequences were detectable in an appreciable proportion (11.9%) of the analyzed plant genomes, we next addressed whether potentially expressed geminiviral sequences could also be detected in plant transcriptomic data. Therefore, using our tBLASTn-based, EGV discovery pipeline, we extended our analyses to 797 assembled *Viridiplantae* transcriptomes (Supplementary Fig. S2) including those from forty-four of the 134 plant species for which genome sequences were screened.

This revealed a total of 112 geminiviral *rep* homologues within thirty-nine of the transcriptomes obtained from twenty-nine distinct angiosperm species (Supplementary Table S2). Of these twenty-nine species, only two (*Capsicum annuum* and *Camellia sinensis*) have ever been previously reported as hosts of geminiviruses (Góngora-Castillo et al. 2012; S. Zhang et al. 2018). Two species (*Solanum melongena* and *C.sinensis*) were found to host EGV sequences when their genome sequence was screened. The other Rep-containing transcripts come from species that are absent from our genome dataset. Interestingly, transcripts with geminiviral *rep* sequence homologues were also found in all other *Camellia* species analyzed (*Camellia chekiangoleosa*, *Camellia oleifera*, *Camellia reticulata*, *Camellia sasanqua*, and *Camellia taliensis*). The highest number of transcripts (*n* = 14) with detectable homology to geminiviral Rep sequences was found in *Diospyros lotus* (Caucasian persimmon).

Overall, EGV Rep homologues were retrieved from forty-two different angiosperm species (Supplementary Table S3), including homologues solely found in thirteen angiosperm genomes, twenty-seven homologues only found in angiosperm transcriptomes, and homologues found in both the genomes and transcriptomes of two angiosperms (*Camellia sinensis* and *Solanum melongena*). This suggests that, as is the case with EGVs in yam (Filloux et al. 2015), the *Camellia* species and *Solanum melongena* contain EGVs that are likely transcriptionally active.

### 3.3 Contextual analysis reveals EGV hotspots and co-transcripts

To better characterize the Rep homologues with the ultimate objective of generating high quality EGV sequence datasets that could be analyzed together with contemporary exogenous geminiviruses, we examined the sequence length distribution of the putative geminivirus-derived Rep proteins recovered from plant genomes and transcriptomes. Using protein-guided alignments with the Exonerate pipeline (Slater and Birney 2005), we identified a set of 2,693 putative Rep protein homologues with a minimum length of ninety amino acids: 2,614 from genomes and seventy-nine from transcriptomes. Due to truncation of some of the potential ORFs encoding Rep proteins, the number of Rep protein homologues (2,693) exceeds that of EGV genomic loci and transcripts combined (2,471). As expected, *Camellia sinensis* showed the highest number of predicted Rep proteins (997 for *Camellia sinensis* var. *assamica* and 1,037 for *Camellia sinensis* var. *sinensis* (Supplementary Table S2)). When comparing the sizes of predicted Rep homologues to those of reference geminivirus Rep sequences, we found that 75 per cent of the endogenous Rep homologues were smaller than 264 aa; the length of the shortest known free-living geminivirus Rep sequence (Supplementary Fig. S3), suggesting that most of the endogenized geminivirus Rep genes could be truncated and/or interrupted by frame shifts and/or stop codons.

**Figure 1.**
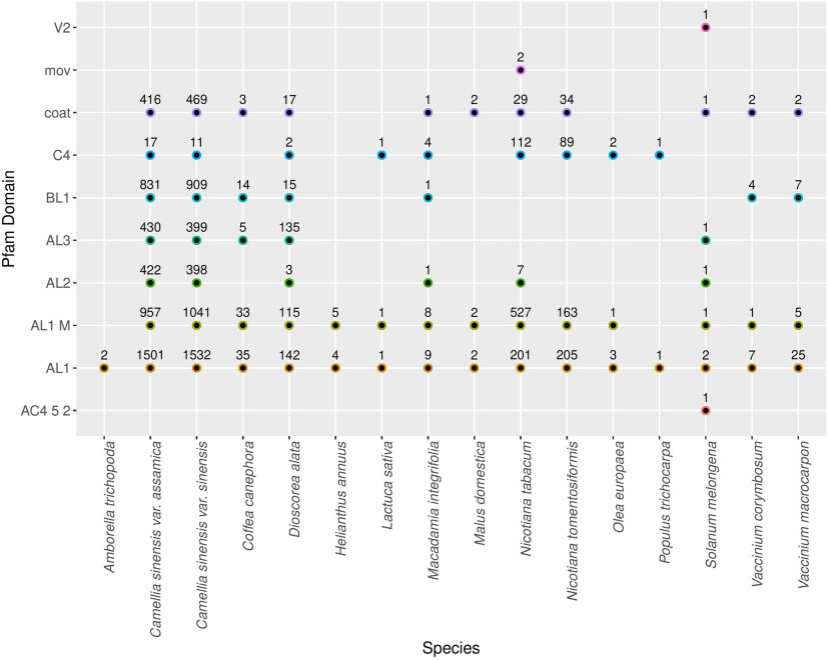
Distribution of predicted core geminivirus Pfam domains within the 5 kb flanking regions of the identified EGVs.

We next addressed the genomic contexts of *rep* homologues in plant genomes and transcriptomes to gain insight into their natures and origins. We first annotated Pfam protein domains within the 2,165 loci spanning 5 kb up- and downstream of Rep hits in plant genomes: referred to as extended genomic loci. Hits were separated into geminivirus and non-geminivirus domains to separately address the completeness of EGVs and their host genomic contexts. As expected, at least one Rep domain (Gemini_AL1, Gemini_AL1_M, or Gemini_AL2) was found in all of the analyzed extended genome loci (Fig. 1). In a significant proportion (1,601/2,165), the extended loci from different plant species were associated with protein domains found in other geminivirus genes (e.g. movement proteins or coat proteins), indicating that many of the geminivirus *rep* homologues likely fall within larger fragments of geminivirus derived sequence (Supplementary Table S4). In *D.alata* and both analyzed *C.sinensis* varieties, we identified potential nuclear shuttle proteins which are characteristic of bipartite begomovirus genomes (Supplementary Table S5). In other cases, most notably in *A.trichopoda*, and *H.annuus*, the *rep* homologues were associated with no other fragments of geminivirus-related DNA.

It is plausible that some of the geminivirus *rep* homologues detected in the nuclear genome and transcriptome datasets could have originated from exogenous geminiviruses that were infecting the analyzed plant samples rather than from EGVs. To ascertain the endogenous status of the *rep* homologues, we first investigated the occurrence of non-geminiviral protein domains around identified EGVs to provide evidence of their integration in host genomes. We found that 882/2,165 (∼41%) of the extended genomic loci contain Pfam domains that are typically found in plant genomes, including those from TEs and host genes, thereby suggesting that a significant proportion of the putative EGVs are indeed endogenous (Supplementary Table S4). While *rep* genes with flanking host protein domains are most certainly endogenous, the absence of such features does not prove that other *rep* genes are not also endogenous as they may have simply been integrated at genome sites that do not encode any protein domains: such as, for example, within TE-derived sequences, tandem repeats, assembly gaps, or genomic dark matter. Alternatively, these other reps could also reside on episomal DNA molecules.

We further examined the density of geminiviral *rep* homologues in the four plant genomes in which they were most abundant (*N.tabacum*, *D.alata*, *C.sinensis var. assamica*, and *C.sinensis var. sinensis*). Contigs from each plant genome assembly were concatenated into a single artificial scaffold and then divided into 100 kb bins within which *rep* hits were counted (Supplementary Fig. S4). Interestingly, we found that *rep* homologues are clustered into hotspots within the *D.alata*, *C.sinensis* var. *assamica*, *C.sinensis* var. *sinensis*, and *N.tabacum* genome with, respectively, up to six, twelve, ten, and seventeen occurrences counted in individual 100 kb bins. This confirms previous reports indicating that EGV sequences are tandemly repeated within the *D.alata* and *N.tabacum* nuclear genomes (Ashby et al. 1997; Bejarano et al. 1996; Filloux et al. 2015; Murad et al. 2004). For the four genomes, the highest number of EGVs observed across genomic contigs is significantly higher than expected if they were randomly distributed (*P*-values < 1.1 × 10^−16^). The organization of EGVs into hotspots therefore seems to be a common feature of plant genomes that contain >100 EGV sequences. It remains to be determined how and why such clusters form.

**Figure 2.**
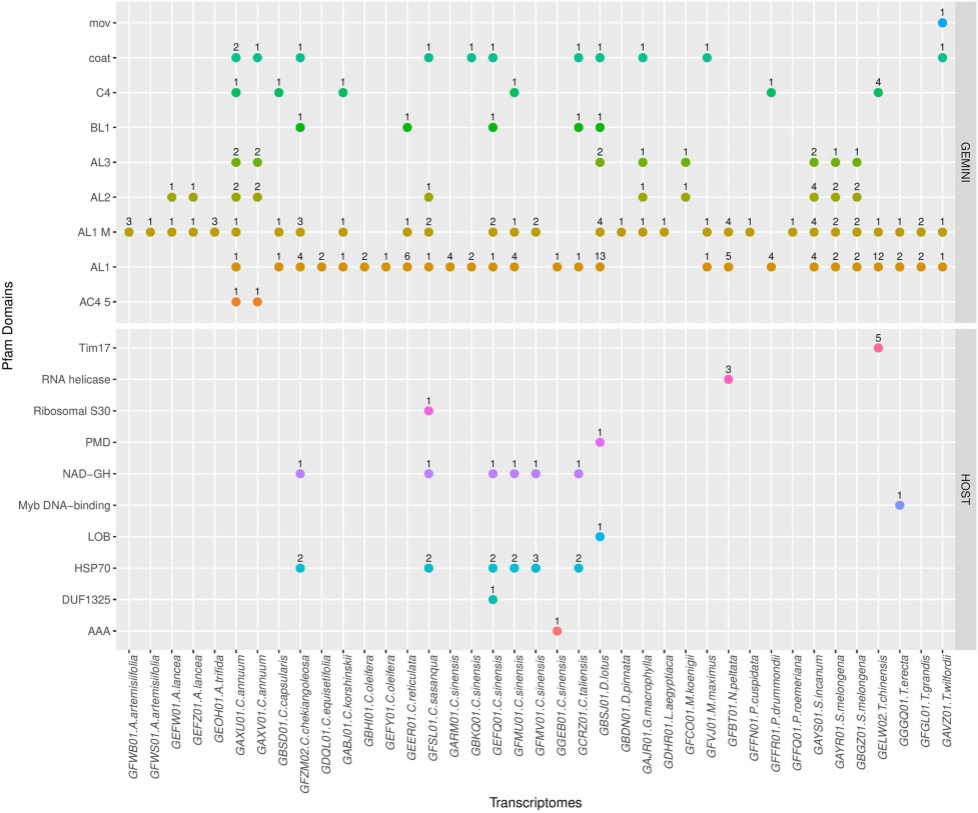
Distribution of core geminivirus and non-geminivirus Pfam domains across the plant transcriptomes around the identified EGVs.

From each transcriptome, protein domains were annotated in the geminivirus Rep homologue-containing transcripts irrespective of whether they were geminivirus or plant protein domains. Besides Rep domains, we detected additional geminiviral domains in Rep-positive transcripts from 21/39 (Supplementary Table S6) of the transcriptomes (Fig. 2). Interestingly, we also detected plant protein domains in Rep-positive transcripts from 10/39 of the transcriptomes (Supplementary Table S6) that probably indicate cotranscripts that have been produced from plant genomic loci. Most notably, six different transcriptomes originating from four *Camellia* species contained transcripts with HSP70 or NAD−GH domains combined with geminiviral domains.

### 3.4 Phylogenetic analysis of EGV Reps reveals novel genus-level geminivirus lineages

We addressed the phylogenetic relationships between the Rep protein sequences of representative exogenous geminiviruses and the EGVs that we had discovered in nuclear genomes and transcriptomes. Rep protein sequences from genomoviruses and phytoplasmal plasmids were used as outgroups. Initial screening retained 2,693 amino acid sequences with best reciprocal hits against geminivirus Rep proteins. To analyze Rep diversity, we first grouped this dataset at the plant species level using a threshold at 80 per cent of amino acid (aa) identity. This resulted in 511 clusters from each of which we selected the longest sequence as a representative of species-level Rep diversity. These sequences were clustered using a 70 per cent aa identity threshold, resulting in 134 clusters. Short length and poorly aligned representative sequences (93 out of 134) were filtered out of this dataset, resulting in a high-quality alignment containing 41 EGV sequences.

**Figure 3.**
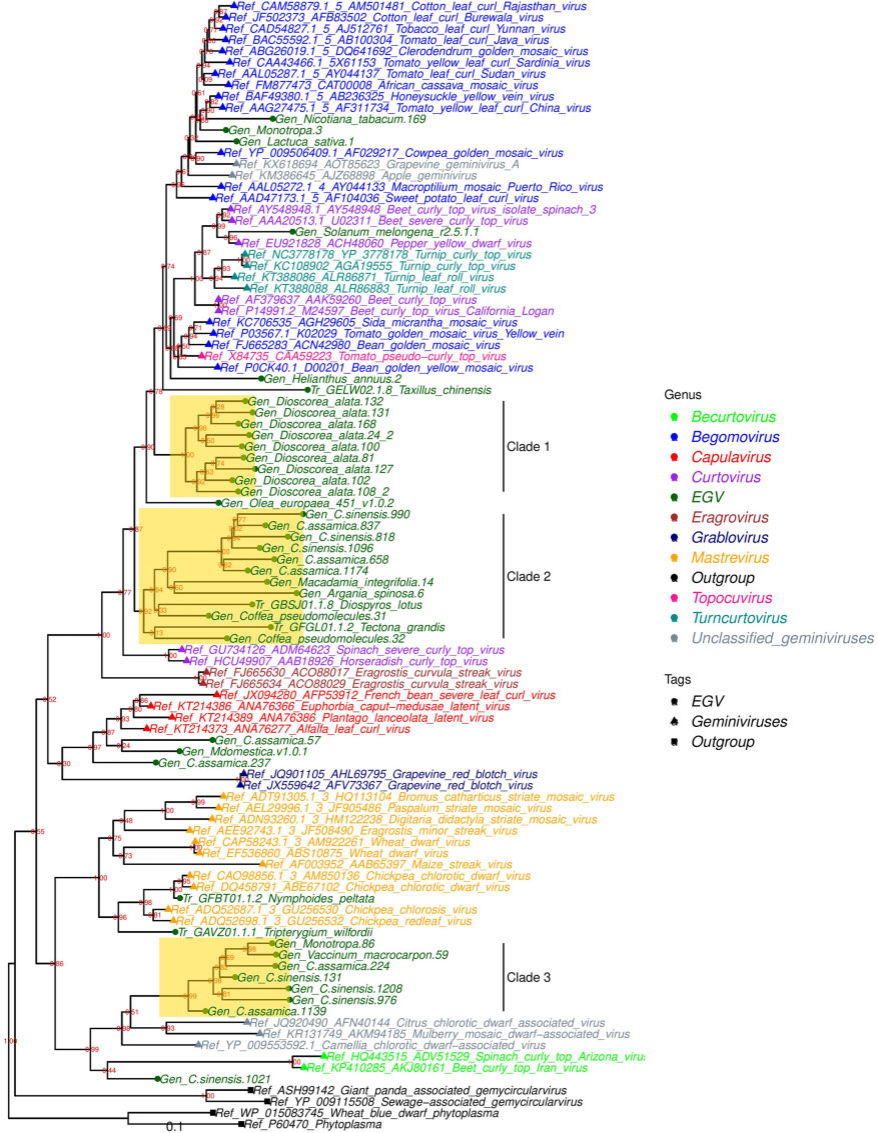
Phylogenetic placement of EGV-encoded Rep proteins. Maximum-likelihood phylogenetic tree constructed using EGV Rep protein sequences retrieved from plant genome sequences (Gen; thirty-six sequences) and transcriptome datasets (Tr; 5 sequences) and from representatives of the geminivirus genus (fifty-five sequences). Distant homologues (four sequences from genomoviruses and phytoplasma) were used as outgroups. Sequences are color-coded according to virus genera, EGV, genomovirus, or phytoplasma origin. Bootstrap values are shown on the nodes of the phylogenetic tree. A scale of substitution rates is provided at the bottom of the tree.

Phylogenetic analysis showed that whereas 8/41 of the EGV Rep sequences within this high quality alignment cluster within the established geminivirus genera, the remainder fall on branches of the phylogeny that are outside these genera (Fig. 3), suggesting that they may represent novel genus-level geminivirus lineages. The EGV Rep sequences clustered within three distinct clades named Clade 1, Clade 2, and Clade 3. Sequences from *C.sinensis* belonged to two of these clades (Clade 2 and Clade 3), suggesting that, as has been reported previously for EGVs in *Dioscorea* and *Nicotiana* species, the EGVs in *C.sinensis* have likely originated from the endogenization of viruses belonging to at least two different geminivirus genera.

Although, according to previous reports, EGV Rep sequences from *Dioscorea* fell within the *Begomovirus* genus, the present phylogenetic analysis places them on a branch that is basal to the begomoviruses, that we refer to here as Clade 1 (Filloux et al. 2015; Murad et al. 2004). Clade 2 branches basal to Clade 1 and the begomovirus. It contains EGV Rep sequences from *C.sinensis*, *Macadamia integrifolia*, *Argania spinose*, and *Coffea* genomes and Rep sequences detected in *Diospyros lotus* and *Tectona grandis* transcriptomes. Clade 3 contains Rep sequences from *Ericales* species (*C. sinensis*, *Vaccinum macrocarpon*, and *Monotropa hypopitys*) and is a sister clade to unassigned geminiviruses that are most closely related to *Citrus chlorotic dwarf associated virus* and *Camellia chlorotic dwarf associated virus.*

### 3.5 Clustering analysis reveals extensive EGV Rep sequence diversity

We applied an all-against-all similarity-based clustering approach to further address the diversity of EGV Rep sequences found in plant genomes and transcriptomes. Contrary to phylogenetic analysis, this approach uses unaligned sequences, which allowed us to analyse all of the 511 species-level representative sequences instead of just the forty-one used for the phylogenetic analysis. We also included fifty-nine Rep proteins from reference geminivirus genomes representing the known breadth of geminivirus Rep diversity. Using the CLANS program (Frickey and Lupas 2004), we found that out of this dataset of 570 Rep proteins, 487 sequences can be clustered into thirty-two groups while eighty-three sequences remain ungrouped (Supplementary Fig. S5 and Table S7). Out of these thirty-two groups, two correspond to the outgroup sequences (genomovirus and phytoplasma) and only seven groups were found to contain known extant geminiviruses. Fourteen out of thirty-two groups contained EGV sequences from the analyzed *C.sinensis* genomes. Out of these fourteen, only three groups contain reference sequences. This suggests an extensive diversity of EGV Rep sequences in the *C.sinensis* genomes and multiple independent geminivirus integration events during the evolutionary history of this species. Likewise, the EGV Rep sequences in *D.alata* fell into three different clusters, also suggesting the occurrence of multiple independent geminivirus integration events in the genome of this species.

### 3.6 EGVs are widespread across the *Ericales*

We observed that EGV Rep homologues are apparently common and abundant in several plant species belonging to different genera of the order *Ericales* including tea plant (genus *Camellia*), cranberry, and blueberry (*Vaccinum* genus) (Supplementary Table S8). To better address the distribution of EGVs in the *Ericales*, we extended our search for EGV Rep homologues to six additional *Ericales* genomes that are available in GenBank (Supplementary Table S9). This screen revealed the presence of EGV Rep homologues in a further three genomes (eighty-seven copies in *Monotropa hypopitys*, fifteen in *Argania spinosa*, and three in *Embelia ribes*) while they were undetected in the genome assemblies for *Actinidia eriantha*, *Actinidia chinensis var. chinensis*, and *Primula veris* (Supplementary Table S9). We further examined this dataset to estimate the number of distinct integration events that may have occurred within plants of the *Ericales* order.

A phylogenetic analysis of the Rep catalytic domain of the geminivirus sequences recovered from the *Ericales* genomes and transcriptomes was therefore performed. By contrast to the previous phylogenetic analysis encompassing well-preserved Rep sequences (Fig. 3), we extended our selection to all the *Ericales* sequences that aligned with the region encoding the catalytic domain of the *rep* gene (i.e. the AL1 domain) (Elmer et al. 1988). Selection of sequences based on the presence of this short region (∼110 amino acids) allowed us to utilize a large fraction of the geminivirus *rep* sequences detected in the analyzed *Ericales* species (1,434 out of 2,108). An alignment of nucleotide sequences encoding the AL1 domain together with seventy four representatives of exogenous geminivirus sequences was used to construct a phylogenetic tree and monophyletic groups of EGV sequences were considered as potentially descending from a single putative ancient integration event.

**Figure 4.**
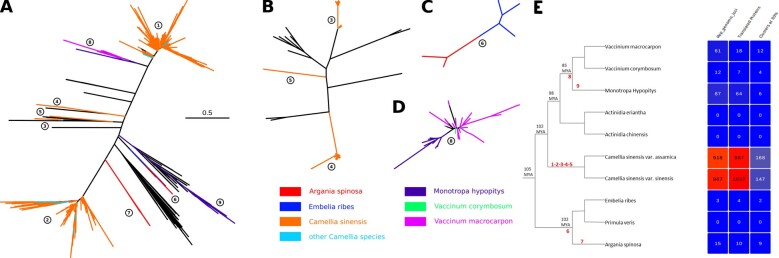
(A) Phylogenetic tree of the *rep* catalytic domain of the sequences discovered within the genomes and transcriptomes of plants in the *Ericales* order along with representative geminivirus sequences focusing on (B) integration event numbers 3, 4, and 5, (C) integration event number 6, and (D) integration event number 8. All trees are constructed at the same scale according to the scale bar of panel A. Phylogenetic trees branches are coloured according to the plant within which the *rep* sequence was discovered (see colour key on bottom right). Branches from *Camellia oleifera*, *Camellia reticulata*, *Camellia sasanqua*, and *Camellia taliensis* have been pooled under ‘other Camellia species’. Geminivirus reference sequences are coloured in black. Integration numbers are indicated with circled numbers (see Supplementary Table S10). (E) The left panel shows a cladogram of the *Ericales* species constructed on the basis of previous work (Rose et al. 2018). Black numbers indicate divergence time estimates (collected from (Li et al. 2013; Rose et al. 2018)) and red numbers indicate integration events. The right panel shows the number of identified viral-like Rep genomic loci and related protein information in *Ericales*.

From this analysis (Fig. 4 and Supplementary Table S10), nine distinct putative integration events were distinguished. Remarkably, while most of the events were only detectable in plants from a single genus (7/9), integration event 6 may predate the divergence of the *Argania* and *Embellia* genera ∼102 mya and event 8 may predate the divergence of the *Monotropa* and *Vaccinum* genera 85 mya (Rose et al. 2018) (see Fig. 4E for details). Note, however, that the times when integration events 6 and 8 occurred could also have been considerably more recent. It remains unclear whether the EGV sequences associated with these events in members of these different plant genera descended from the same integrated sequence. For example, in the case of event 6 there is no evidence in *Primula* assembly of homologous EGV sequences to those found in *Argania* and *Embellia* and they are also absent from raw sequencing reads. A single integration origin for the *Argania* and *Embellia* EGV sequences would imply that close relatives of these sequences should be present in *Primula* spp. Their absence in *Primula* spp. implies one of the following scenarios: 1, that the integration event was ancient and the resulting EGV sequences were deleted in the Primula lineage but not in the other two, 2, that the integration was more (perhaps much more) recent than ∼102 mya and the resulting EGV sequences were transferred between the *Argania* and *Embellia* lineages by hybridization, or 3, that the *Argania* and *Embellia* EGV sequences were each derived from independent integrations of similar viruses. Determining which of these scenarios is most plausible would require detailed investigation of the loci at which these EGV sequences were integrated so as to determine if they are indeed all descended from a single common ancestral geminivirus.

Importantly, whereas a minimum of five independent integration events were predicted to have occurred within the genomes of members of the *Camellia* genus, none of these events were detectable in the analyzed members of any of the other genera of the *Ericales* order. Sequences of each of the five distinct *Camellia* EGV lineages were found in *Camellia sinensis*, while EGV sequences from only three of the five lineages were found in the other analyzed *Camellia* species. Although all the EGV sequences from these other *Camellia* species were retrieved from transcriptome and not genome datasets, these ‘non-*sinensis*’ sequences nevertheless cluster closely together with *C.sinensis* EGV sequences, indicating that they may descend from geminivirus sequences that integrated into the genome of an ancestral *Camellia* species prior to its differentiation into the species analyzed here.

### 3.7 Assembly of putative EGV integrons from *Camellia sinensis* genomes

Of all the plant genomes assessed in this study, that of *Camellia sinensis var. sinensis* displayed the highest EGV copy numbers and the largest degree of EGV diversity. We attempted to reconstruct complete genomes from representatives of the original integrating viruses, hereafter called EGV integrons, for the two main *Camellia* EGV lineages: i.e. those carrying Clade 2 or Clade 3 *rep* genes. After experiencing difficulties with alignment-based approaches, we directly indexed and assembled batches of k-mers found in the extended loci. All but one of the sequence assemblies which were of approximately geminivirus genome length (i.e. between 2.5 and 5 kb), did not appear to represent complete geminivirus-like genomes. The complete assembly had 19x sequencing coverage, contained a Clade 3 *rep* gene, had a size of 4.6 kb and, and displayed a complete set of geminivirus-like genes. This putative EGV integron is clearly related to the geminiviruses, based on the presence of a geminivirus-like coat protein gene, a putative movement protein gene, *rep*A and *rep*B homologues, and a characteristic geminivirus-like virion strand origin of replication nonanucleotide sequence, 5′-TAATATTAC-3′ (Supplementary Fig. S6).

A complementary phylogenetic analysis confirmed that the *rep* of this putative EGV integron clusters within Clade 3, a sister group of three extant geminiviruses that are presently unassigned to a genus, namely *Citrus chlorotic dwarf associated virus* (CCDaV), *Mulberry mosaic dwarf associated virus* (MMDaV), and *Camellia chlorotic dwarf-associated virus* (CaCDaV) (Zhang et al. 2018) (Supplementary Fig. S7). In addition, phylogenetic analysis of geminivirus coat protein sequences revealed that the coat protein of the putative EGV integron clusters with those of grabloviruses (data not shown). These results suggest that the *Camellia*-associated geminivirus from which the putative EGV integron originated was not a direct ancestor of contemporary CaCDaV.

### 3.8 EGVs from *Camellia* spp. are transcriptionally active and may be functionally expressed

The analysis of six different transcriptomes obtained from four *Camellia* species (*C.chekiangoleosa*, *C.sasanqua*, *C.sinensis*, and *C.taliensis*) provided evidence of Rep-encoding Clade 3 EGV sequences being co-transcribed with HSP70 domains (Fig. 2). Using these co-transcript sequences as query in BLASTn searches on the TSA sequence database from GenBank, we identified similar co-transcripts in four other *C.sinensis* transcriptomes.

**Figure 5.**
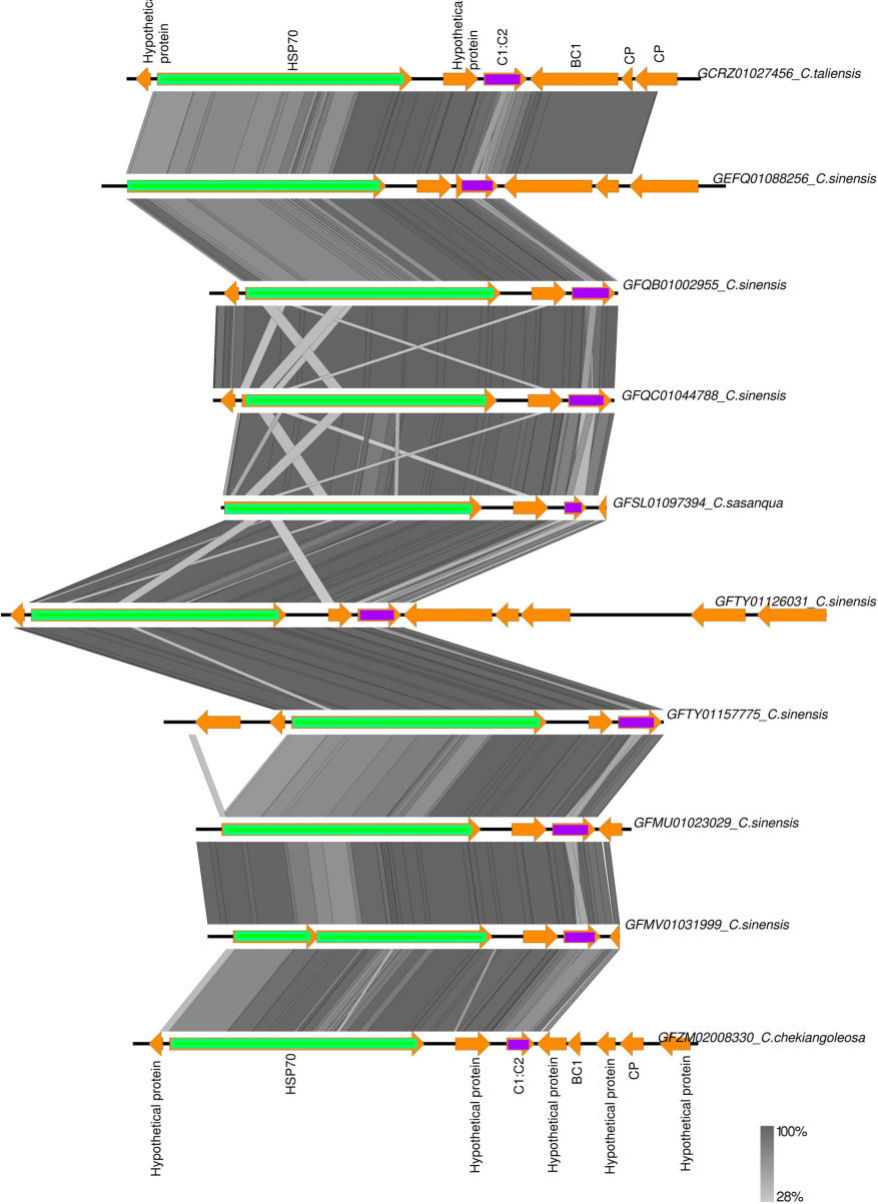
Gene synteny comparison between transcripts from different *Camellia* species. Vertical grey lines and blocks show the similarity between the transcript sequences based on tBLASTx. Genes encoded by the *Camellia* transcripts have been displayed using arrows (hypothetical protein; CP: coat protein; BC1: movement protein; C1: C2: replication-associated protein).

We compared the sequences and structures of ten such Rep-HSP70 transcripts using tBLASTx (Fig. 5). All contain highly conserved HSP70 genes in the same orientation relative to the Clade 3 EGV *rep* genes, supporting a common integration origin for these ten *rep* genes. In addition, these co-transcripts were produced from multiple genomic loci in each individual plant genome, suggesting that the genomic region encompassing both rep-HSP70 genes was probably duplicated in the plant genome after the initial *rep* integration.

We addressed the direction of natural selection in the identified HSP70 genes based on the ratio of non-synomymous (dN) versus synonymous (dS) substitution rates: dN/dS ratios significantly above 1 indicate potential positive selection, ratios equal to 1 reflect neutral evolution and ratios significantly below 1 indicate potential purifying selection (Jeffares et al. 2014). dN/dS, analysis using HSP70 sequences from four different *Camellia* species (*C.taliensis*, *C.sinensis*, *C.sasanqua*, and *C.chekiangoleosa*), indicated that twenty-two sites were evolving under significant purifying selection and none were detectably evolving under positive selection (Supplementary Table S11). Similarly, *rep* genes from the same transcripts also exhibit at least four sites apparently evolving under significant purifying selection, and none evolving under significant positive selection (Supplementary Table S12).

Together, the conserved structure, transcription, and purifying selection acting on a subset of the potentially amino acid encoding sites of the Rep-HSP70 sequences suggests a functional role for Rep-HSP70 co-transcripts in *Camellia*. Interestingly, comparative sequence analyses using BLASTn mapped at least two transcripts (GFQC01044788.1, GFQB01002955.1) with high similarity (query coverage: = 100%, per cent of identity: >99.8%) to their corresponding genomic loci in the genome of *Camellia sinensis* var. *sinensis.* This high degree of similarity further supports the possibility that the integrated EGV-Rep sequences are transcriptionally active and may be functionally expressed. In addition, these results suggest that the initial *rep* integration nearby a HSP70 gene may have predated the divergence of the *Camellia* species about 12.5 mya (Li et al. 2013) and that the Rep-HSP70 duplication events have likely occurred intermittently with *Camellia* speciation events as has been suggested for EGV sequences in *Dioscorea* (Filloux et al. 2015) and *Nicotiana* (Murad et al. 2004) species.

### 3.9 DNA methylation analysis of EGVs in *Camellia sinensis var. assamica*

DNA methylation of cytosines is associated with transcriptional silencing of repetitive and foreign DNA (Pelizzola and Ecker 2011). The public availability of *Camellia sinensis* var. *assamica* DNA methylome obtained by bisulfite sequencing (Wang et al. 2019) provided an opportunity to determine whether the EGV sequences in *C.sinensis* might be epigenetically regulated.

**Figure 6.**
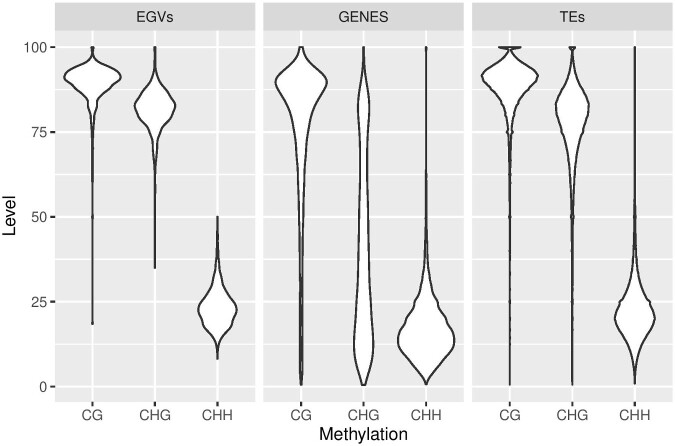
Violin plot representing the distributions of average cytosine methylation (CHH, CHG, and CG) levels of protein-coding genes, EGV Rep genomic loci, and repeated genes in the *Camellia sinensis* var. *assamica* genome.

As we observed from our transcriptome analysis, most of the EGV loci in *Camellia sinensis* are possibly transcriptionally silent under the conditions addressed here. Specifically, we investigated cytosine methylation levels of EGVs, genomic repeats including TEs, and protein-coding genes in different sequence contexts (CG, CHG, and CHH) (Fig. 6). Observing each genomic context separately revealed a statistically significant difference in the methylation levels between EGVs and genes in all contexts (*P* < 2 × 10^−16^ in CG, CHG, and CHH contexts using a pairwise Wilcoxon test). By contrast, the comparison between EGVs and TEs revealed a significant difference for the CHG (*P* = P < 2 × 10^−16^) and CHH contexts (*P* = 7.5 × 10^−15^) but not for the CG context (*P* = 0.11). Consistent with previous findings (Wang et al. 2019), cytosine methylation levels of EGVs were more similar to those of TEs and other genomic repeats and significantly higher than those of protein-coding genes; i.e. EGVs displayed high methylation levels in the CG, CHG, and CHH contexts. The high methylation level of EGVs in *Camellia sinensis* var. *assamica* is consistent with most EGV loci being silenced in this variety. This is reminiscent of previous results in *N.tabacum* where high EGV methylation levels were also observed (Kovařík et al. 2000; Murad et al. 2004). It is likely that the observed degree of EGV methylation would be associated with transcriptional gene silencing via the RNA-dependent DNA methylation (RdDM) pathway which relies on the activity of a set of proteins that produce small-interfering RNAs (siRNAs) homologous to target loci and mediate epigenetic modifications of chromosomes at these target loci. Therefore, the hypothetical possibility remains that transcribed EGVs such as those found in *Camellia* species may help defend plants against infections by cognate viruses, should they still be extant, by maintaining a cellular pool of anti-geminivirus siRNA that would target and silence the gene expression of incoming viruses.

## Supplementary data

Supplementary data are available at *Virus Evolution* online.

## Authors contributions

V.S. performed analyses and analyzed the data with help from P.L.; V.S. and F.M. drafted the manuscript and all authors contributed to the final version; F.M. conceived the study.

**Conflict of interest:** None declared.

## Supplementary Material

veaa071_Supplementary_Data
