# Supplementary figures and images for: Large-scale survey reveals pervasiveness and potential function of endogenous geminiviral sequences in plants
Source: Virus Evol. 2020 Sep 21;6(2):veaa071. doi: 10.1093/ve/veaa071 (PMC7758297; doi:10.1093/ve/veaa071)

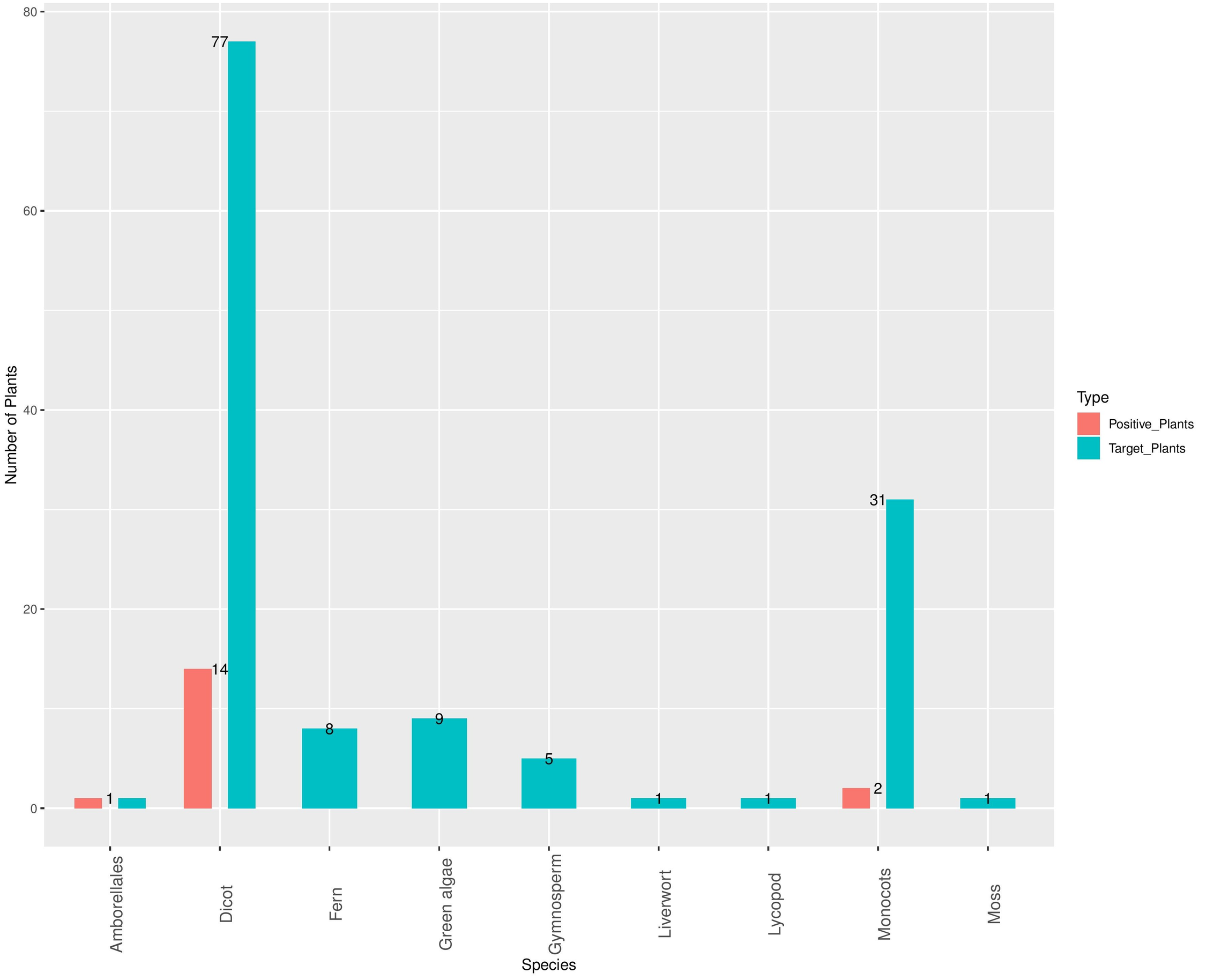

Supplement: veaa071_Supplementary_Data [file veaa071_supplementary_data.zip › Figure S1.tif]

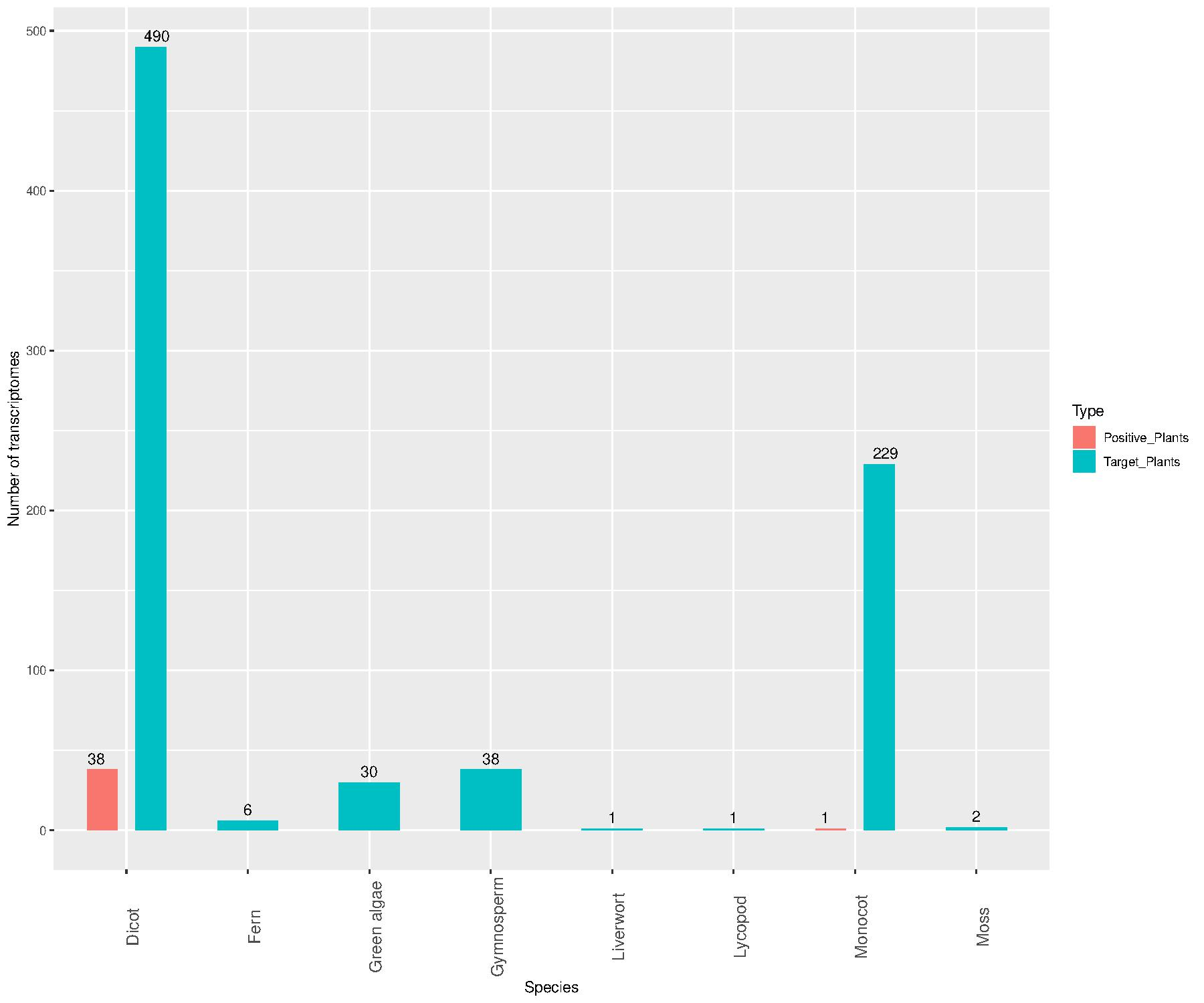

Supplement: veaa071_Supplementary_Data [file veaa071_supplementary_data.zip › Figure S2.tif]

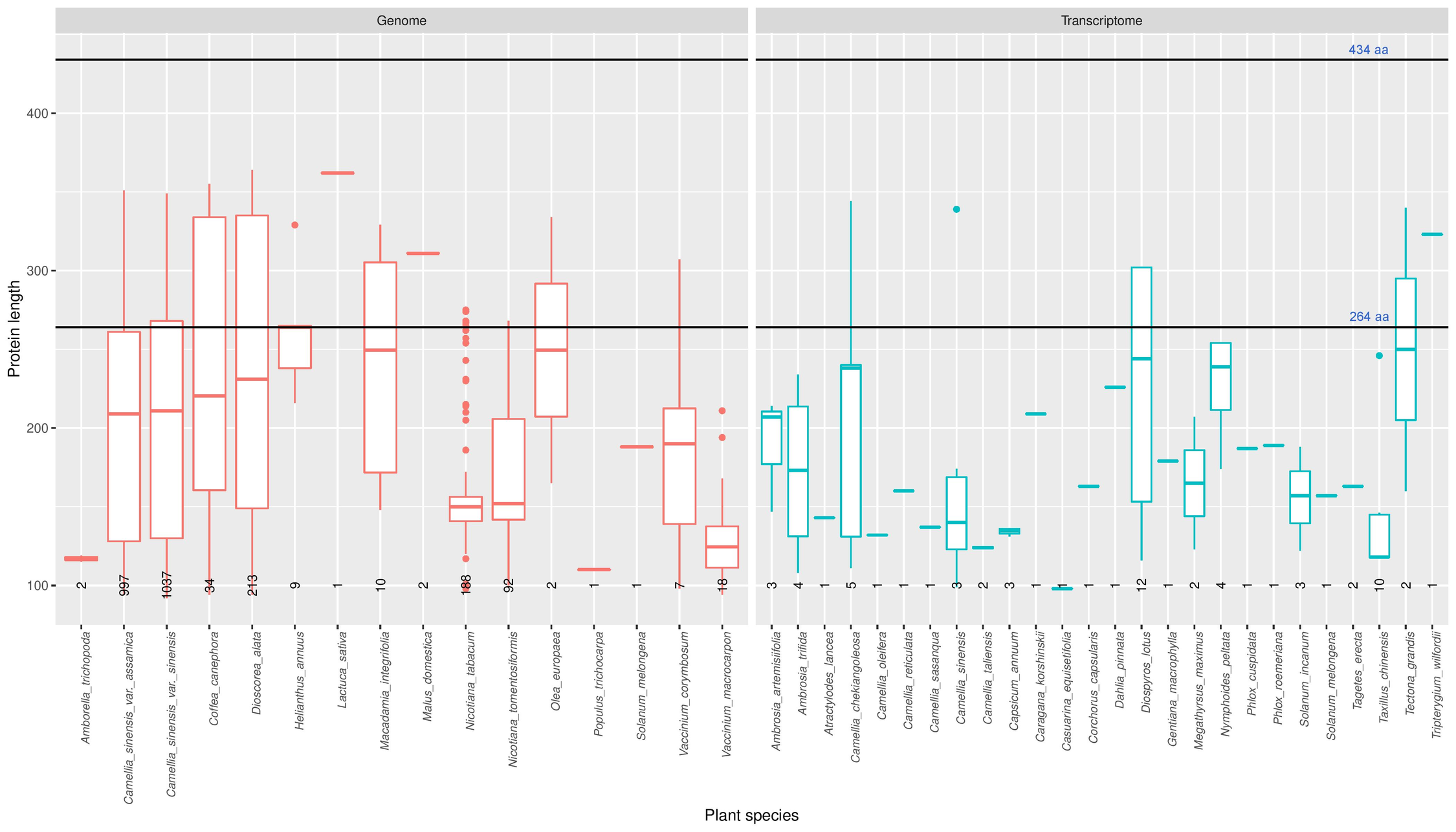

Supplement: veaa071_Supplementary_Data [file veaa071_supplementary_data.zip › Figure S3.tif]

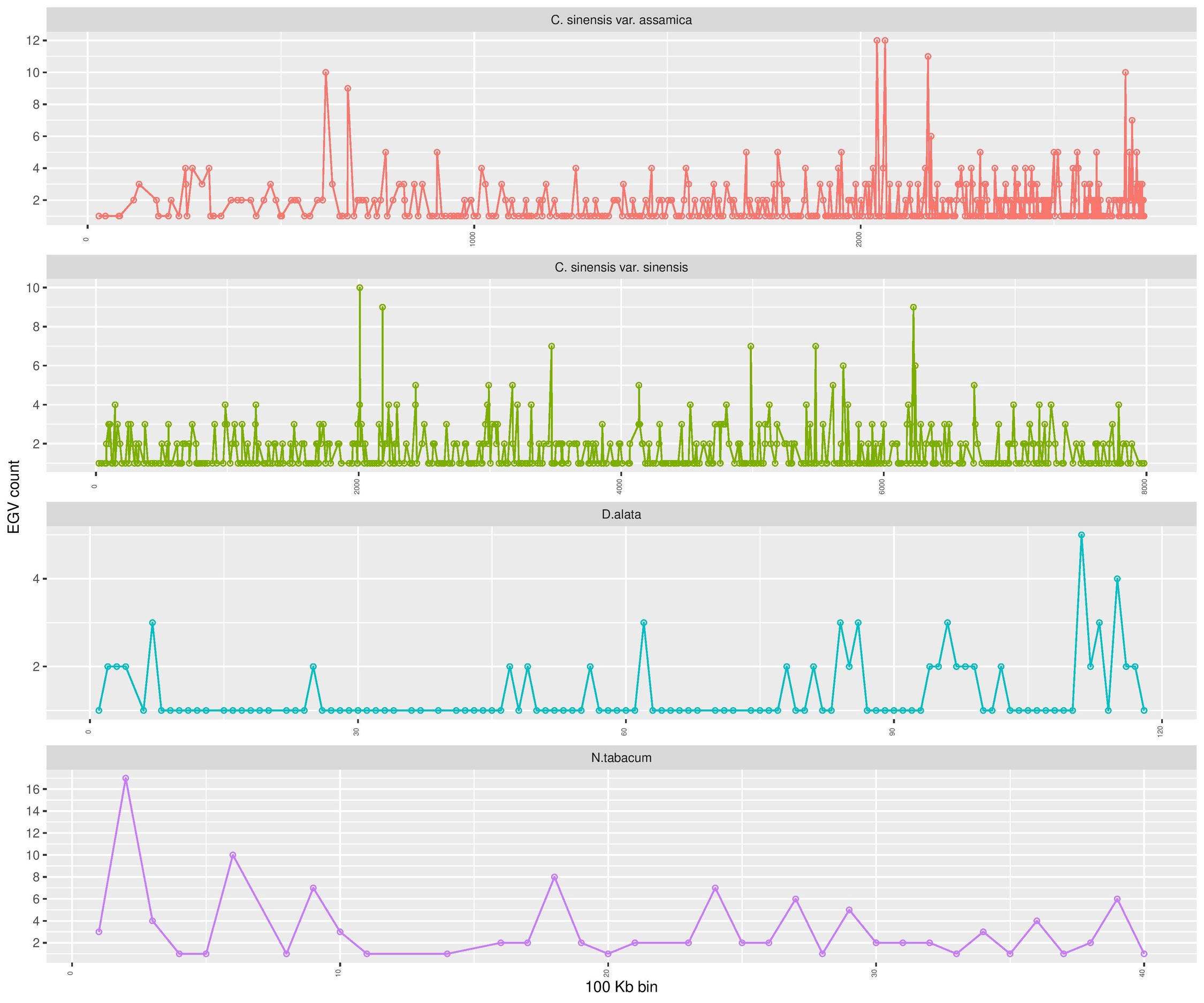

Supplement: veaa071_Supplementary_Data [file veaa071_supplementary_data.zip › Figure S4.tif]

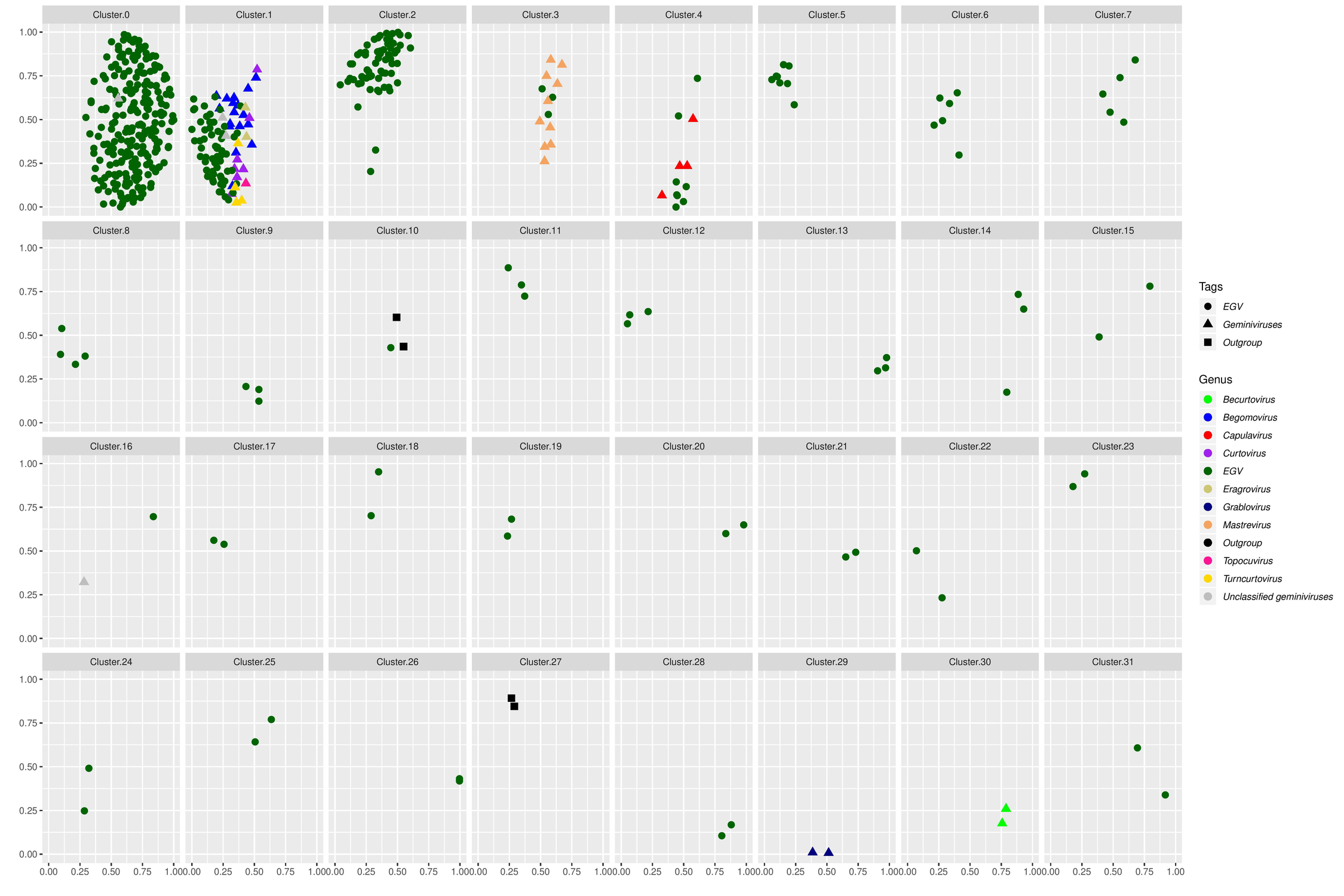

Supplement: veaa071_Supplementary_Data [file veaa071_supplementary_data.zip › Figure S5.tif]

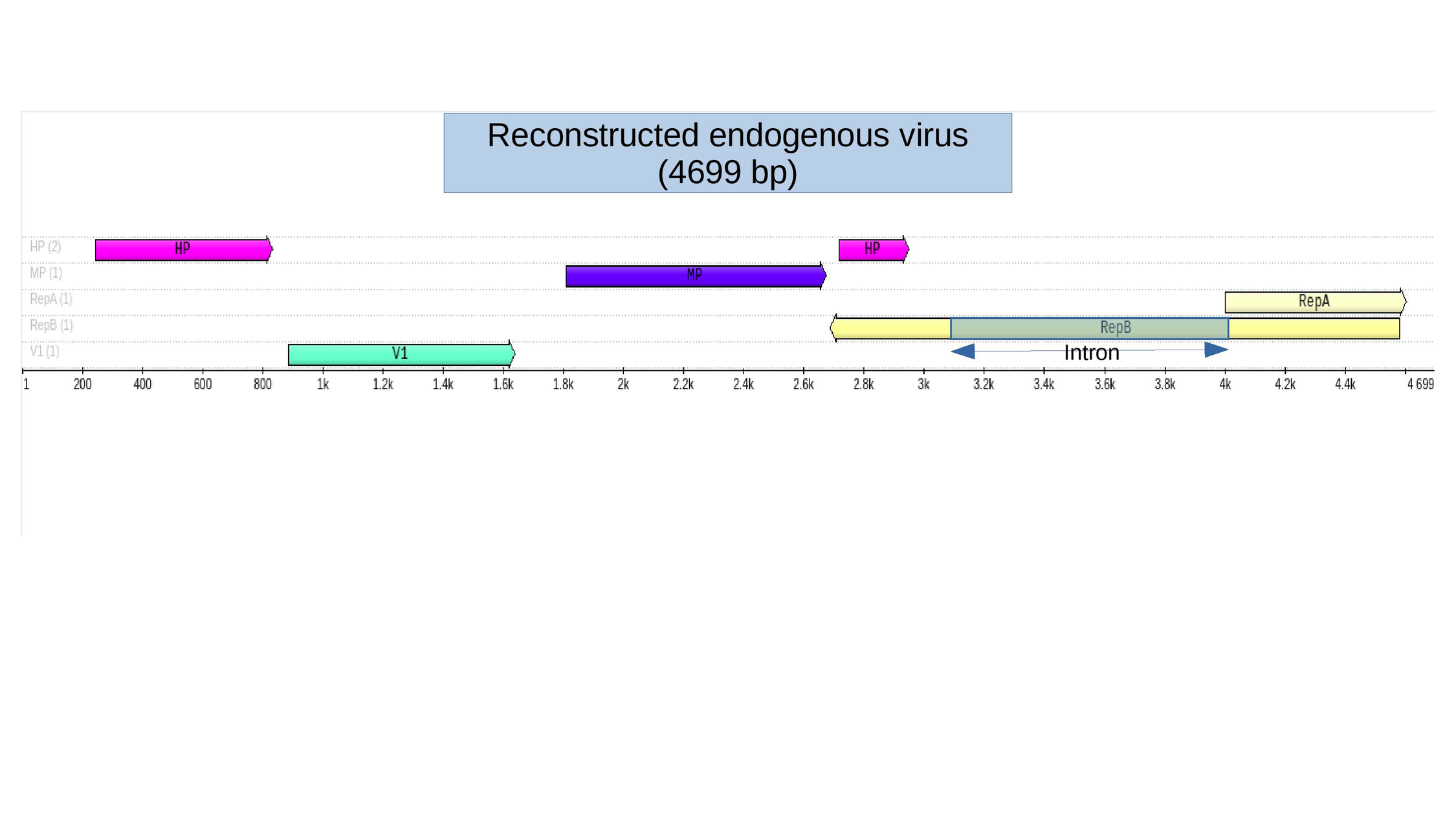

Supplement: veaa071_Supplementary_Data [file veaa071_supplementary_data.zip › Figure S6.tif]

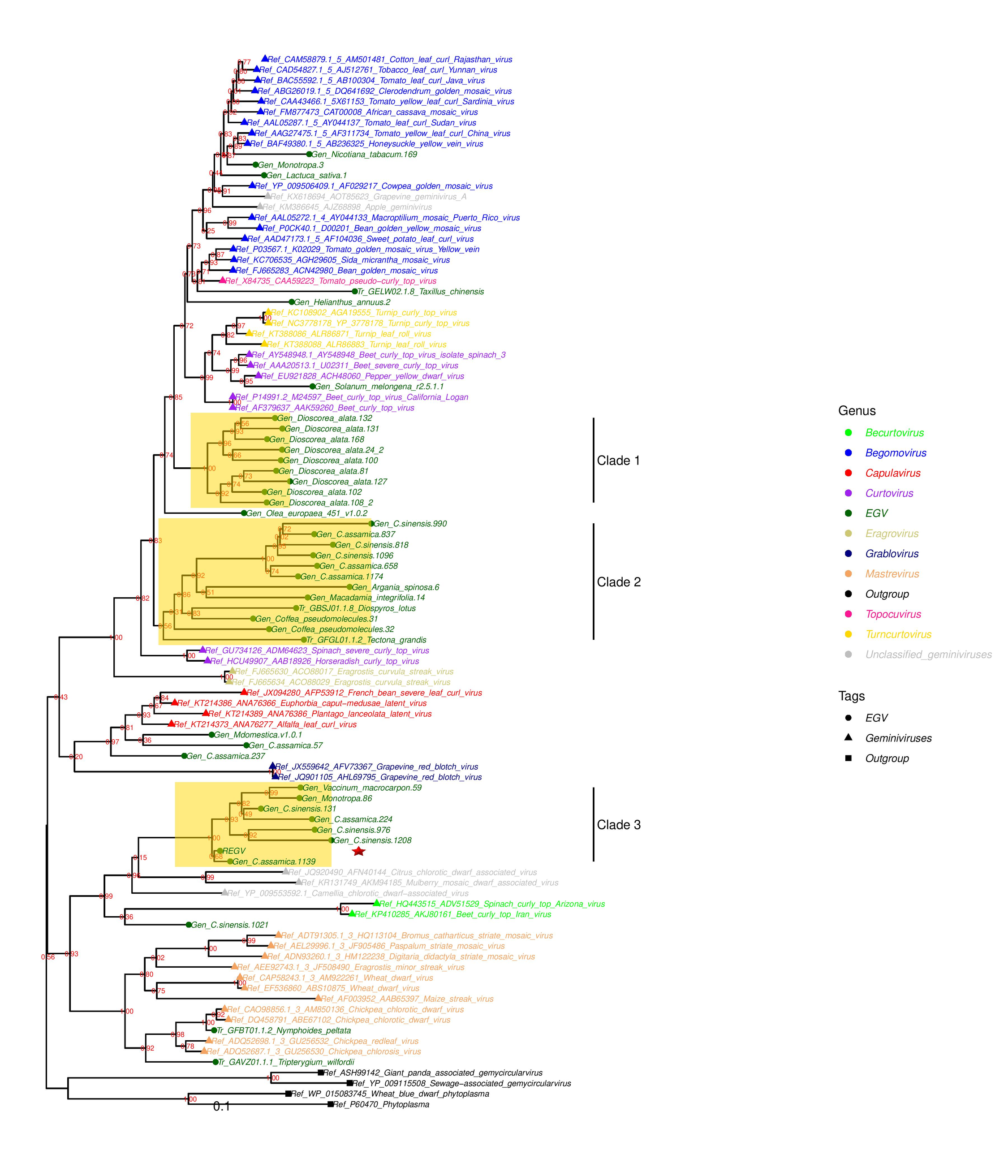

Supplement: veaa071_Supplementary_Data [file veaa071_supplementary_data.zip › Figure S7.tif]
